# The relationship between latent profile-based physical exercise and academic burnout in college students: the mediating roles of self-control and problematic smartphone use

**DOI:** 10.3389/fpsyg.2026.1876915

**Published:** 2026-07-01

**Authors:** Feifei Zhang, Yuanwen Geng, Qinqin Lin

**Affiliations:** 1 Department of Physical Education, Handan University, Handan, Hebei, China; 2 School of Physical Education, Yanshan University, Qinhuangdao, China

**Keywords:** academic burnout, latent profile analysis, physical exercise, problematic smartphone use, self-control

## Abstract

**Introduction:**

Academic burnout is a prevalent issue among college students, yet research on its relationship with physical exercise from a person-centered perspective remains limited. This study aimed to identify latent profiles of physical exercise among college students and to examine the mediating roles of self-control and problematic smartphone use in the association between these exercise profiles and academic burnout.

**Methods:**

A questionnaire survey was conducted among 722 Chinese college students. Latent profile analysis and bootstrap mediation analysis were employed to analyze the data.

**Results:**

The results revealed three distinct exercise profiles: occasional exercisers (27.9%), developing exercisers (31.7%), and regular exercisers (40.3%). Significant differences were found across the three profiles in self-control, problematic smartphone use, and academic burnout. Specifically, regular exercisers reported the highest self-control and the lowest levels of problematic smartphone use and academic burnout, followed by developing exercisers, with occasional exercisers showing the least favorable outcomes. Mediation analyses indicated that self-control significantly mediated the relationship between both the developing and regular exercise profiles (compared to occasional exercisers) and academic burnout. Moreover, self-control and problematic smartphone use acted as sequential mediators in these relationships. However, problematic smartphone use alone did not show a significant mediating effect.

**Discussion:**

These findings highlight the heterogeneity in college students’ physical exercise patterns and suggest that interventions aimed at reducing academic burnout should consider promoting regular physical exercise, which appears to be associated with higher self-control and subsequently lower problematic smartphone use. Tailored strategies targeting different exercise profiles may be more effective in addressing academic burnout.

## Introduction

Academic burnout is a common negative learning attitude and behavior among students, primarily characterized by emotional exhaustion, reduced academic efficacy, and detachment from learning (Hu and Schaufeli, 2009). It can occur from elementary school through university, but is especially prevalent on college campuses. Research indicates that over half of university students experience moderate or higher levels of academic burnout (Xie and Xiao, 2022). Studies have found that academic burnout is associated with lower academic performance, reduced self-esteem, diminished learning engagement, and impaired well-being (Chen, 2016). Furthermore, it shows associations with future job burnout after graduation (Cao et al., 2024). Therefore, understanding the correlates and patterns of academic burnout among university students is important for promoting their career development and enhancing the quality of the future workforce.

Physical exercise refers to leisure-time physical activities primarily aimed at health maintenance, recreation, and enjoyment, which can be measured by frequency, intensity, and duration (Zhang et al., 2025). It is associated with lower levels of negative emotions and behaviors, such as depression and anxiety among college students (Philippot et al., 2022). Given that depression and anxiety frequently co-occur with academic burnout (Liu et al., 2024), physical exercise is related to lower academic burnout, and this relationship may accompany reduced affective issues. However, previous research has primarily adopted a variable-centered approach to examine the relationship between physical exercise and academic burnout (Ye et al., 2024), overlooking inter-individual heterogeneity. In other words, college students may exhibit different latent profiles of physical exercise due to individual differences. To date, few studies have taken a person-centered approach to identify these latent profiles, and the relationship between such profiles and academic burnout remains underexplored. Therefore, this study intends to use latent profile analysis to investigate the heterogeneity of physical exercise among college students. Subsequently, we will treat the identified latent profiles as independent variables to explore their relationship with academic burnout and differences in underlying patterns. Accordingly, this study proposes Hypothesis H1: Heterogeneous latent profiles of physical exercise exist among college students, and these profiles differ in their associations with academic burnout.

Self-control is the process of inhibiting or overcoming one’s own desires and needs, thereby changing ingrained or habitual behaviors and ways of thinking—essentially replacing one behavior or thought pattern with another (Tan and Guo, 2008). College students with strong self-control tend to display patience and persistence, along with positive academic characteristics (Lin et al., 2025). Studies have shown a significant negative correlation between self-control and academic burnout (Luo et al., 2020; Du et al., 2025); lower self-control is often associated with higher levels of academic burnout (Luo et al., 2020; Du et al., 2025). Additionally, research indicates a positive relationship between physical exercise and self-control. For instance, exercise volume has been found to correlate positively with self-control (Du et al., 2025). In a 2- to 4-month self-control training program for college students, participants showed significant improvement in self-regulation during the latter two-month regulated exercise phase compared to the first two-month control phase (Oaten and Cheng, 2006). Accordingly, this study proposes Hypothesis H2: Self-control shows a mediating association in the relationship between latent profiles of physical exercise and academic burnout.

Problematic smartphone use refers to maladaptive behaviors characterized by excessive or uncontrolled preoccupation with smartphone use (Lopez-Fernandez et al., 2014). Research has identified it as a significant predictor of academic burnout (Zhang et al., 2021). For instance, excessive smartphone use is negatively associated with college students’ academic performance (Qaisar et al., 2017), and smartphone dependence or addiction is associated with poor time management, which is related to higher academic burnout (Sadeghi Bimorgh et al., 2023). Problematic smartphone use is associated with academic burnout and also varies with physical exercise (Ji et al., 2024), consistent with the principle that “Exercise is Medicine” (Thompson et al., 2020). Regular physical exercise is positively associated with self-control, which in turn is negatively associated with reliance on smartphone use. Accordingly, a significant negative correlation has been found between physical exercise and symptoms of smartphone dependence (Shi et al., 2025). Thus, this study proposes Hypothesis H3: Problematic smartphone use shows a mediating association in the relationship between latent profiles of physical exercise and academic burnout.

Empirical research shows that self-control is significantly associated with problematic smartphone use (Jiang and Zhao, 2016). Surveys on smartphone dependence among college students indicate that individuals with poorer self-control tend to report higher levels of smartphone dependence, using smartphones more frequently and intensively (Pan et al., 2023). Other studies suggest that higher self-control is associated with lower smartphone-dependent procrastination (Wang et al., 2025). Multiple studies have confirmed that self-control is negatively associated with problematic smartphone use (Jiang and Zhao, 2016; Mao et al., 2022). Thus, the patterns linking physical exercise to academic burnout may involve a sequential pathway from internal factors (e.g., self-control) to external factors (e.g., problematic smartphone use). Furthermore, the relationships between physical exercise and self-control (Du et al., 2025) and between problematic smartphone use and academic burnout (Zhang et al., 2021) have been empirically supported. Therefore, this study further proposes Hypothesis H4: Self-control and problematic smartphone use serve as sequential mediators in the relationship between latent profiles of physical exercise and academic burnout among college students.

## Methods

### Participants

Using cluster sampling, a questionnaire survey was conducted in March and April 2026 among first-, second-, and third-year undergraduate students from five universities in Jiangxi, Anhui, Liaoning, Guizhou, and Hubei provinces. Before the survey, researchers explained the study and obtained informed consent from all participants. The final valid sample consisted of 722 students (301 males, 41.7%; 421 females, 58.3%), after excluding invalid responses based on repetitive answers, reverse-coded items, and completion time. Among participants, 355 (49.2%) were from rural areas, and 367 (50.8%) were from urban areas; 222 (30.8%) were freshmen, 351 (48.6%) were sophomores, and 149 (20.6%) were juniors. The most recent physical fitness test scores were: ≤60 (92, 12.8%), 61–70 (273, 37.8%), 71–80 (175, 24.2%), 81–90 (124, 17.2%), and 91–100 (58, 8%). All participants provided written informed consent.

### Measures

#### Academic Burnout Scale

The Chinese version of the College Student Academic Burnout Scale revised by Lian et al. (2005) was used. It consists of 20 items rated on a 5-point Likert scale from 1 (strongly disagree) to 5 (strongly agree). Higher total scores indicate greater academic burnout. In this study, the Cronbach’s *α* coefficient was 0.786. The scale showed good reliability among college students.

#### Self-Control Scale

The Self-Control Scale for college students revised by Tan and Guo (2008) was used, comprising 8 items. Each item is rated on a 5-point scale from 1 (strongly disagree) to 5 (strongly agree). Higher total scores indicate greater self-control. In this study, the Cronbach’s α coefficient was 0.855. The scale showed good reliability among college students.

#### Problematic Smartphone Use Scale

The Problematic Smartphone Use Scale revised by Zhang et al. (2019) was used, comprising 10 items. Each item is rated on a 5-point Likert scale from 1 (strongly disagree) to 5 (strongly agree). Higher total scores indicate greater problematic smartphone use. In this study, the Cronbach’s *α* coefficient was 0.923. The scale demonstrated good reliability among college students.

#### Physical Exercise Rating Scale

The Physical Exercise Rating Scale developed by Liang (1994) was used. It assesses college students’ physical exercise across three dimensions: intensity, frequency, and duration. Physical exercise level is calculated as intensity × (duration - 1) × frequency. Each dimension is rated on a 5-point scale, yielding total scores from 0 to 100. The test–retest reliability of the scale is 0.82 (Liang, 1994). In this study, the Cronbach’s α coefficient was 0.719. The scale showed good reliability among college students.

### Data analysis

First, descriptive statistics and correlation analysis were performed using SPSS 26.0. Second, latent profile analysis (LPA) of physical exercise was conducted using Mplus 8.3. The resulting profiles were used as independent variables, and the BCH command was applied to test for significant differences across profiles in self-control, problematic smartphone use, and academic burnout. Third, with the physical exercise profiles as the independent variable, self-control and problematic smartphone use as mediators, and academic burnout as the dependent variable, a bootstrap-based mediation analysis for multicategorical independent variables was conducted using an SPSS macro.

## Results

### Common method bias control and testing

To control for potential common method bias, the survey was administered anonymously, and reverse-scored items were used. Harman’s single-factor test was conducted to examine common method bias. The KMO and Bartlett’s sphericity tests were performed on the data, yielding a KMO value of 0.933 and a Bartlett’s test value of 18484.290.55 (df = 1,128, *p* < 0.01), indicating that the data were suitable for factor analysis. An unrotated exploratory factor analysis was conducted on all items, extracting eight factors with eigenvalues greater than 1. The variance explained by the largest factor was 26.50% (<40%). Therefore, common method bias was not a serious concern in this study.

### Descriptive statistics and correlational analysis

The descriptive and correlational results (see Table 1) showed that physical exercise was significantly positively correlated with self-control and significantly negatively correlated with problematic smartphone use and academic burnout. Self-control was significantly negatively correlated with both problematic smartphone use and academic burnout, while problematic smartphone use was significantly positively correlated with academic burnout. Additionally, gender, grade, major, residence, annual income, and physical fitness test scores were significantly correlated with some study variables. Therefore, these variables were included as controls.

**Table 1:** Descriptive statistics and correlations for all variables.

Variables	*M*	*SD*	1	2	3	4	5	6	7	8	9	10
1. Gender	1.58	0.49	1									
2. Residence	1.49	0.50	−0.022	1								
3. Grade	1.90	0.71	−0.025	0.101**	1							
4. Major	2.96	1.48	0.087*	0.060	−0.027	1						
5. PF	2.70	1.14	−0.162**	0.075*	0.129**	−0.035	1					
6. IH	2.42	1.19	0.109**	−0.337**	0.001	−0.020	0.017	1				
7. PE	24.18	23.38	−0.278**	0.011	0.127**	0.120**	0.295**	0.041	1			
8. SC	59.83	9.26	0.012	0.073*	0.008	0.010	−0.020	0.012	0.187**	1		
9. PSU	28.81	8.33	0.101**	−0.073*	−0.008	−0.005	−0.117**	−0.017	−0.205**	−0.592**	1	
10. AB	43.41	7.19	−0.061	0.065	−0.006	0.023	0.020	−0.135**	−0.200**	−0.602**	0.438**	1

^*^p < 0.05, ^**^p < 0.01, ^***^p < 0.001; PF, Physical fitness test; IH, Income of households; PE, physical exercise; SC, self-control; PSU, problematic smartphone use; AB, academic burnout.

### Latent profile analysis of physical exercise

**Figure 1:**
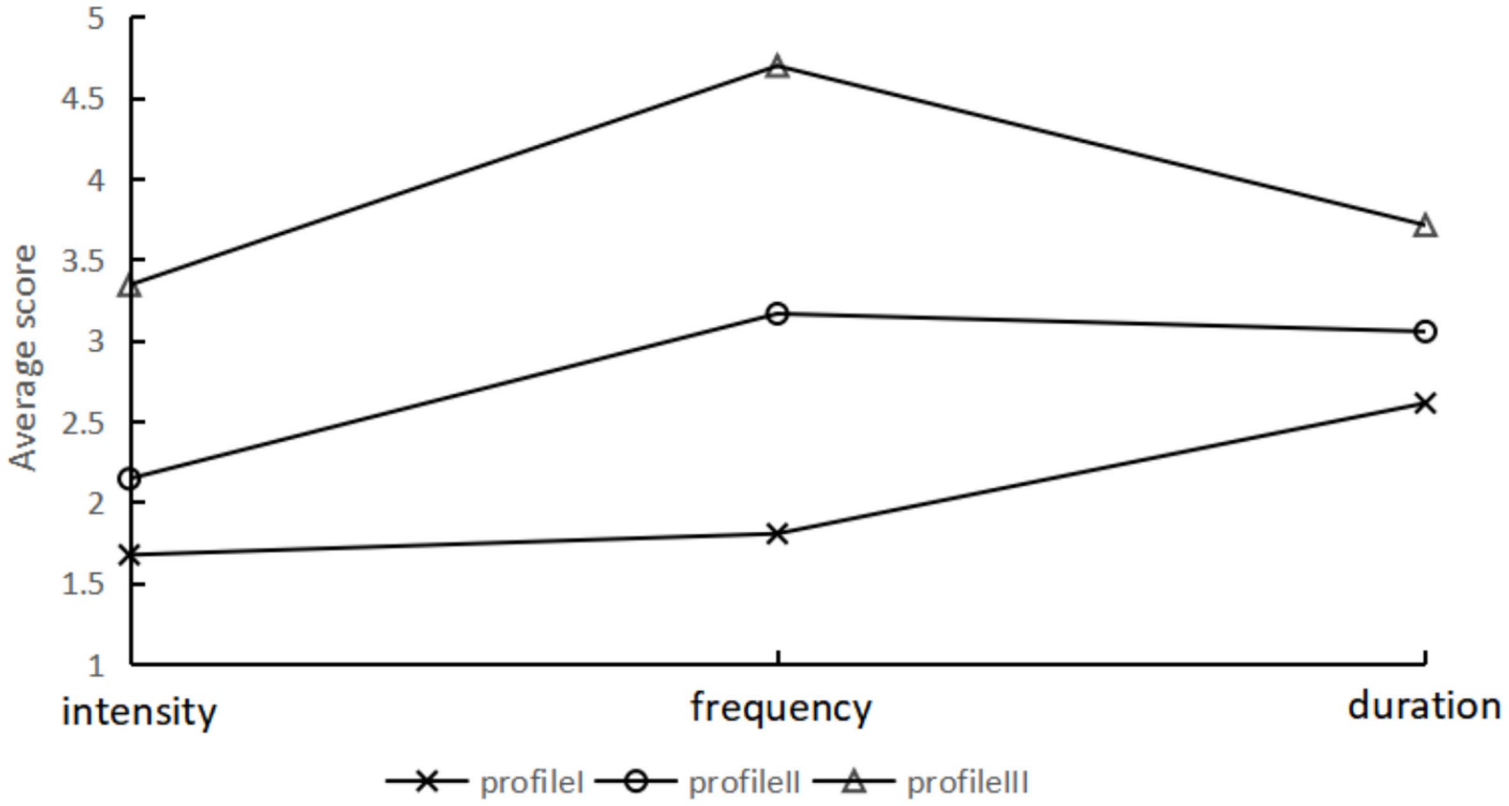
Three latent profiles of physical exercise.

As shown in Table 2, AIC, BIC, and aBIC values consistently decreased as the number of profiles increased. Entropy values for the first four profiles exceeded 0.80, indicating good classification accuracy. Model fit improved from one to four profiles. The LMR tests for the first four profiles were all significant. Specifically, both the three- and four-profile models showed significant LMR (*p* = 0.0000) and BLRT (*p* = 0.0000) results, indicating significantly better fit than the first two profiles. However, the three-profile model had a higher Entropy value than the four-profile model. Considering AIC, BIC, aBIC, Entropy, LMR, and BLRT, the three-profile model was selected as optimal. Based on item response patterns (Figure 1), the naming of the three profiles was guided by prior LPA studies on physical exercise (Ling et al., 2022) and the observed patterns of average scores for intensity, frequency, and duration. Specifically: occasional exercisers scored near baseline on all three indicators; developing exercisers exhibited moderate levels; regular exercisers scored consistently high on all dimensions. The three latent profiles were labeled as follows: (1) Occasional exercisers (27.9%, profile I)—mild intensity, short duration, frequency of 2–3 times per month; (2) Developing exercisers (31.7%, profile II)—progressing from low to moderate intensity and short to moderate duration, with a frequency of 1–2 times per week; (3) Regular exercisers (40.3%, profile III)—moderate-to-high intensity, moderate-to-long duration, frequency of 3–5 times per week, meeting the definition of regular exercise.

**Table 2:** Model fit information for latent profile analysis of physical exercise.

Model	Log(L)	AIC	BIC	aBIC	Entropy	pLMR	pBLRT	Profile probabilities
One-profile	−3405.012	6822.024	6849.517	6830.465	—	—	—	—
Two-profile	−3165.377	6350.755	6396.575	6364.822	0.801	0.0010	0.0000	0.55/0.45
Three-profile	−3115.647	6259.294	6323.443	6278.989	0.839	0.0000	0.0000	0.28/0.40/0.32
Four-profile	−3066.828	6169.657	6252.133	6194.978	0.800	0.0000	0.0000	0.31/0.13/0.33/0.23
Five-profile	−3043.246	6130.492	6231.296	6161.440	0.798	0.1837	0.0000	0.20/0.18/0.16/0.13/0.33

### Differences in outcome variables across latent profiles of physical exercise

Using the three identified latent profiles, the BCH command was applied to examine differences in self-control, problematic smartphone use, and academic burnout across profiles (see Table 3). Significant differences were found for all three outcome variables. For self-control, the order of means was Profile III (Regular exercisers) > Profile II (Developing exercisers) > Profile I (Occasional exercisers), with all pairwise differences significant. For problematic smartphone use, the order was Profile I > Profile II > Profile III, with all pairwise differences significant. For academic burnout, the order was Profile I > Profile II > Profile III, also with all pairwise differences significant.

**Table 3:** Between-group differences in outcome variables across latent profiles of physical exercise.

Variables	Latent profiles	Mean	SE	Differences between groups (class K-class K + i, i = 0, 1, 2, 3)			Overall chi-square test
				I	II	III	
SC	Profile I	56.899	0.469	0			39.477***
	Profile II	59.903	0.611	35.152***	0		
	Profile III	61.812	0.688	13.941***	3.873*	0	
PSU	Profile I	31.638	0.549	0			37.818***
	Profile II	28.680	0.582	37.006***	0		
	Profile III	26.953	0.545	12.578***	4.205*	0	
AB	Profile I	46.069	0.432	0			48.664***
	Profile II	43.386	0.473	46.672***	0		
	Profile III	41.572	0.501	16.144***	6.217*	0	

^*^p < 0.05, ^**^p < 0.01, ^***^p < 0.001; SC, self-control; PSU, problematic smartphone use; AB, academic burnout.

### Testing the mediating associations of self-control and problematic smartphone use in the relationship between latent profiles of physical exercise and academic burnout

Controlling for gender, residence, grade, major, physical fitness test scores, and income, a bootstrap method (5,000 resamples) was used to test the multicategorical mediation model, with latent profiles of physical exercise as the independent variable, self-control and problematic smartphone use as mediators, and academic burnout as the dependent variable. Results showed that compared to occasional exercisers, both developing and regular exercisers had significantly negative direct associations with academic burnout. After adding self-control and problematic smartphone use as mediators, the direct association of regular exercisers (vs. occasional exercisers) with academic burnout remained significant, whereas the direct association of developing exercisers became non-significant. Additionally, self-control was negatively associated with academic burnout, while problematic smartphone use was positively associated with it. Regarding associations with mediators, compared to occasional exercisers, both developing and regular exercisers were significantly positively associated with self-control, but neither was significantly associated with problematic smartphone use (see Table 4).

**Table 4:** Regression results for the mediation model.

Dependent variable	Independent variable	Fit indices		Coefficient significance	
		*R^2^*	*F*	*β*	t
AB		0.089	8.736***	—	—
	Developing exercisers	—	—	−2.460***	−3.662
	Regular exercisers	—	—	−4.861***	−7.271
	Gender	—	—	−1.396**	−2.553
	Residence	—	—	0.206	0.373
	Grade	—	—	0.174	0.471
	Major	—	—	0.284	1.601
	PF	—	—	0.453	1.914
	IH	—	—	−0.630**	−2.717
AB		0.414	50.180***	—	—
	SC	—	—	−0.389***	−13.792
	PSU	—	—	0.112***	3.573
	Developing exercisers	—	—	−0.995	−1.826
	Regular exercisers	—	—	−2.351***	−4.258
	Gender	—	—	−1.188**	−2.686
	Residence	—	—	1.052*	2.363
	Grade	—	—	0.024	0.080
	Major	—	—	0.221	1.552
	PF	—	—	0.267	1.390
	IH	—	—	−0.519**	−2.785
SC		0.056	5.310***	—	—
	Developing exercisers	—	—	2.987***	3.391
	Regular exercisers	—	—	5.307***	6.052
	Gender	—	—	0.836	1.166
	Residence	—	—	1.757*	2.430
	Grade	—	—	−2.272	−0.563
	Major	—	—	−0.148	−0.635
	PF	—	—	−0.601	−1.936
	IH	—	—	0.207	0.680
PSU		0.379	48.339***	—	—
	SC	—	—	−0.522***	−19.087
	Developing exercisers	—	—	−1.131	−1.743
	Regular exercisers	—	—	−1.201	−1.828
	Gender	—	—	1.483**	2.825
	Residence	—	—	−0.529	−0.998
	Grade	—	—	0.251	0.710
	Major	—	—	−0.030	−0.174
	PF	—	—	−0.741***	−3.254
	IH	—	—	−0.164	−0.738

Occasional exercisers served as the reference group. *p < 0.05, **p < 0.01, ***p < 0.001; PF, Physical fitness test; IH, Income of households; SC, self-control; PSU, problematic smartphone use; AB, academic burnout.

**Figure 2:**
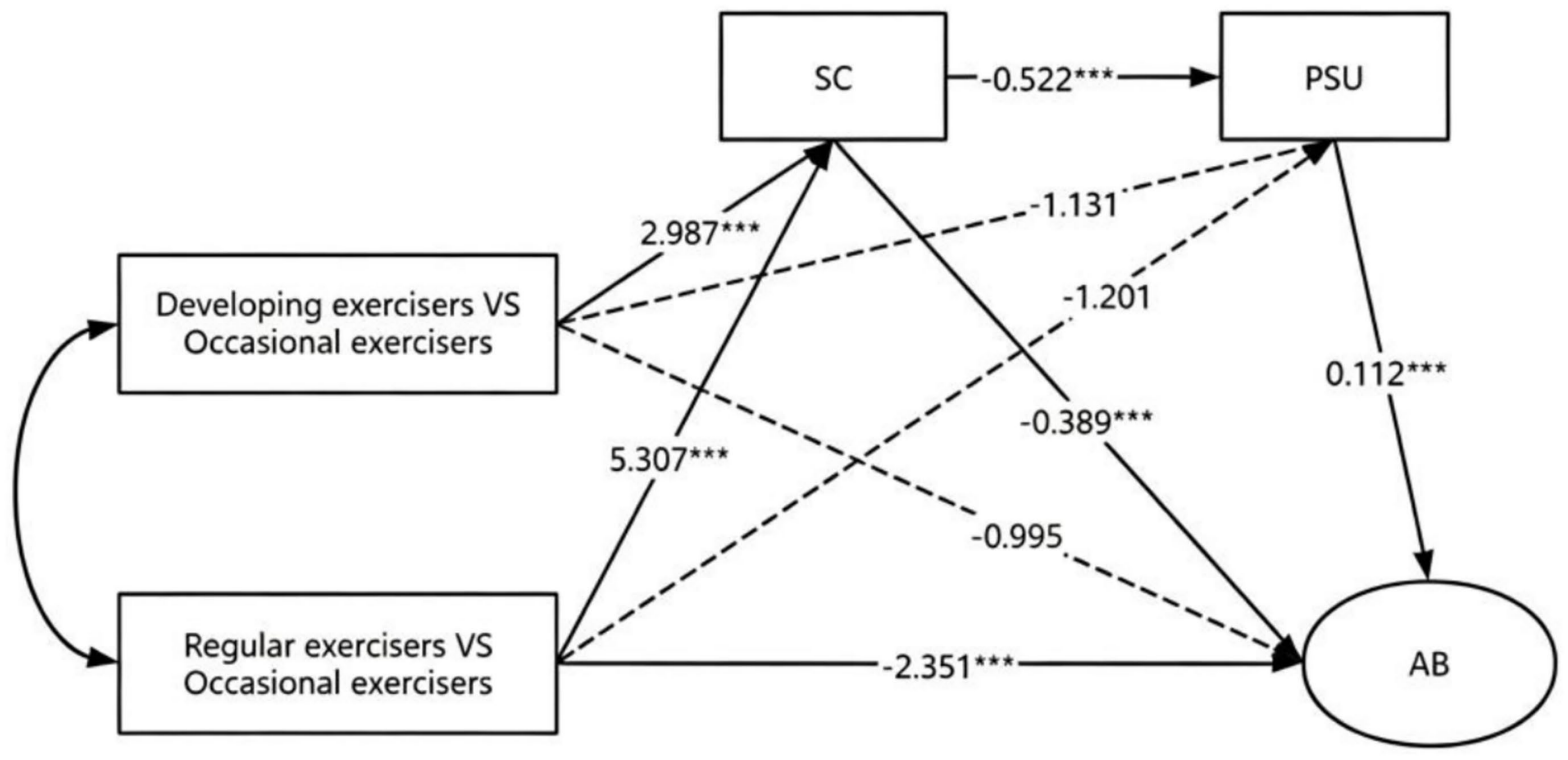
Path model for mediation effect testing. ***p < 0.001.

Compared to occasional exercisers, self-control showed a significant mediating association between the developing exerciser profile and academic burnout (95% CI [−1.763, −0.584]), with an indirect effect of −1.162, and also between the regular exerciser profile and academic burnout (95% CI [−2.830, −1.354]), with an indirect effect of −2.065. However, problematic smartphone use did not show a significant mediating association for either the developing exerciser profile (95% CI [−0.329, 0.009]) or the regular exerciser profile (95% CI [−0.354, 0.008]) with academic burnout. Furthermore, self-control and problematic smartphone use showed a significant serial mediating association between the developing exerciser profile and academic burnout (95% CI [−0.343, −0.047]), with an indirect effect of −0.175, and also between the regular exerciser profile and academic burnout (95% CI [−0.566, −0.095]), with an indirect effect of −0.311. Meanwhile, the developing exerciser profile did not show a significant direct association with academic burnout (95% CI [−2.065, 0.075]), whereas the regular exerciser profile did (95% CI [−3.434, −1.267]). See Table 5 and Figure 2 for details.

**Table 5:** Mediation analysis of SC and PSU in the relationship between latent profiles of PE and AB.

Profile	Mediator	Effect	SE	95% CI	
				Lower	Upper
Developing exercisers	SC	−1.162	0.302	−1.763	−0.584
	PSU	−0.127	0.088	−0.329	0.009
	SC → PSU	−0.175	0.075	−0.343	−0.047
Regular exercisers	SC	−2.065	0.383	−2.830	−1.354
	PSU	−0.135	0.093	−0.354	0.008
	SC → PSU	−0.311	0.120	−0.566	−0.095

Occasional exercisers served as the reference group. SC, self-control; PSU, problematic smartphone use.

## Discussion

This study adopted a person-centered approach to conduct a latent profile analysis of college students’ physical exercise. The results revealed heterogeneity in exercise patterns, identifying three distinct latent categories: occasional exercisers, developing exercisers, and regular exercisers. These three groups differed significantly in their primary sources of exercise behavior. Specifically, occasional exercisers scored significantly lower than the other two groups in exercise intensity, duration, and frequency; developing exercisers showed moderate levels; and regular exercisers scored the highest on all three indicators. This distribution suggests that differences in college students’ physical exercise are not randomly distributed but are closely related to the degree of behavioral habituation. Notably, developing and regular exercisers together accounted for over 70% of the sample, indicating that most college students have a certain foundation in physical exercise or are at a critical stage of habit formation (Qiu et al., 2020), which provides a favorable basis for university-based exercise interventions. Although occasional exercisers represented a smaller proportion, their low intensity, low frequency, and short duration place them near a sedentary state, marking them as a key group requiring attention. These findings support the use of a person-centered perspective to understand college students’ exercise behavior and provide empirical evidence for developing stratified and differentiated strategies to promote physical exercise (Ling et al., 2022).

The results also showed that, compared with occasional exercisers, both regular and developing exercisers exhibited higher self-control, lower problematic smartphone use, and lower academic burnout, which is largely consistent with previous findings (Du et al., 2025; Ji et al., 2024; Shi et al., 2025). First, regular physical exercise is positively associated with individuals’ conscious regulation of behavior and self-control (Yu et al., 2025). Muraven’s (2010) study demonstrated that aerobic exercise is significantly associated with higher self-control: participants who engaged in aerobic or endurance training after a period of sedentary behavior showed marked improvements in self-control. Second, related research has found that various types of exercise interventions are associated with reduced smartphone dependence among college students (Pirwani and Szabo, 2025). From a neurobiological perspective, physical exercise is involved in modulating dopamine and its bidirectional receptor regulation (Wei, 2023), thereby showing associations with reduced behavioral issues such as smartphone dependence and internet addiction in adolescents. Finally, physical exercise is associated with greater feelings of pleasure, comfort, and fulfillment among college students, contributing to an overall positive psychological state (Tian and Yang, 2024). These positive experiences are associated with lower levels of low mood and academic pressure (Tian and Yang, 2024), and thus are related to lower academic burnout. In summary, physical exercise is positively associated with self-control and negatively associated with problematic smartphone use and academic burnout, suggesting that parents, teachers, and schools should consciously enhance college students’ physical exercise levels to support their positive physical and mental development.

This study found that after including self-control and problematic smartphone use as mediators, college students in the regular exercise type still showed significantly lower levels of academic burnout compared to the occasional exercise type, and this difference remained robust after controlling for demographic variables such as gender, grade, and major. These findings further confirm the central role of regular physical exercise in academic promotion patterns, supporting the main effect model of exercise (Xu, 2025), which posits that physical exercise is directly associated with lower academic burnout. Mediation analysis revealed that, compared to the occasional exercise type, self-control showed a significant mediating association between both the developing and regular exercise types and academic burnout (Du et al., 2025), indicating that physical exercise is associated with lower academic burnout through its positive association with college students’ self-control (Xu, 2025). This also suggests that physical exercise is associated with better regulation of behavior and emotions in learning and other activities. Therefore, schools and teachers should actively encourage college students, especially those with lower self-control, to engage in physical exercise (Du et al., 2025). Additionally, teachers can promote in-class physical activities to enhance students’ self-control, which is associated with lower academic burnout.

Notably, compared with occasional exercisers, problematic smartphone use did not show a significant standalone mediating association between either developing or regular exercisers and academic burnout. This non-significant finding warrants careful interpretation from multiple perspectives. First, the relationship between physical exercise and problematic smartphone use may be indirect, requiring other psychological variables such as self-control to fully manifest (Heidari et al., 2025). When self-control is statistically accounted for, the unique variance explained by problematic smartphone use as a mediator may be substantially reduced (Qin et al., 2020). Second, the serial mediation pathway (self-control → problematic smartphone use) was significant for both developing and regular exercisers, suggesting that problematic smartphone use may function more as a downstream consequence of low self-control than as an independent mediator. In other words, physical exercise is associated with higher self-control, and higher self-control is associated with lower problematic smartphone use, which in turn is associated with lower academic burnout. Without the self-control pathway, problematic smartphone use alone does not significantly transmit the association between exercise and burnout. Third, measurement issues may have contributed. Our self-report scale assessed perceived dependence on smartphone use, but objective indicators such as screen time, app usage frequency, notification logs, or time spent on specific activities (e.g., social media, gaming) might capture different facets of problematic use. Some researchers argue that problematic use is context-dependent; thus, global self-report measures may fail to capture situational variability. Fourth, the relatively high level of self-control among regular and developing exercisers may have suppressed the mediating role of problematic smartphone use, as self-control may act as a protective factor that attenuates the negative associations of smartphone use. Future research should integrate objective behavioral data and experimental designs to further validate the robustness of this pathway.

Furthermore, serial mediation analysis revealed that, compared with occasional exercisers, self-control and problematic smartphone use formed a significant serial mediating pathway between both developing and regular exercisers and academic burnout. This finding indicates that the indirect associations between physical exercise and academic burnout involve multiple pathways: physical exercise is positively associated with self-control, which in turn is negatively associated with problematic smartphone use (Kim et al., 2017), and lower problematic smartphone use is further associated with lower academic burnout (Kim et al., 2017). This serial pathway reflects a progressive pattern from “higher internal psychological resources” to “lower external behavioral problems,” offering strong theoretical integration. Notably, the serial mediation association was significant for both developing and regular exercisers, whereas the standalone mediating association of problematic smartphone use was not. This further suggests that problematic smartphone use does not independently show an association with academic burnout but rather is associated with negative outcomes primarily under conditions of lower self-control. In other words, self-control is a key node in the pattern linking problematic smartphone use to academic burnout; only when self-control is low is problematic smartphone use more strongly associated with academic burnout (Kardefelt-Winther, 2014). Physical exercise is associated with a disruption of this risk pattern through its positive association with self-control. These results highlight that in university mental health and physical exercise interventions, emphasis should be placed on cultivating students’ self-control—particularly among those with low levels of exercise—through physical exercise, which is associated with reduced problematic smartphone use and lower academic burnout (Zhang et al., 2021).

### Implications

From an intervention perspective, the observed associations suggest several practical considerations. Universities could design tiered physical activity programs based on students’ exercise profiles. For occasional exercisers, low-threshold activities such as walking groups, short guided workouts, or gamified exercise challenges may help initiate behavior change without requiring high initial motivation. For developing exercisers, structured routines with peer support, scheduled group activities, or integration of exercise into daily schedules (e.g., active commuting, standing breaks during classes) could reinforce habit formation. For regular exercisers, variety and autonomy in exercise choices, along with opportunities for leadership (e.g., leading group activities), may sustain long-term engagement.

Importantly, self-control training could be integrated into physical education curricula. For example, goal-setting exercises, self-monitoring of progress, and reflection on behavioral choices may enhance self-regulatory skills that generalize beyond exercise contexts. Digital wellness modules addressing mindful smartphone use could complement exercise interventions, particularly for students with lower self-control. These modules might include education on notification management, scheduled screen-free periods, and strategies for replacing smartphone use with physical activity.

Given that non-significant standalone mediation of problematic smartphone use does not imply its irrelevance, interventions should target self-control as a primary mechanism, with problematic smartphone use as a secondary behavioral outcome. School policies that restrict smartphone use during class time or designate smartphone-free zones on campus may also support the observed associations. However, as all findings are correlational, these practical recommendations should be implemented alongside rigorous evaluation to determine their effectiveness.

### Limitations

This study explores the relationships among physical exercise, self-control, problematic smartphone use, and academic burnout, offering insights for promoting college students’ academic performance. However, several limitations should be noted. First, the cross-sectional design precludes determining the dynamic developmental trajectories among the variables. Future research should combine longitudinal and experimental methods to clarify dynamic changes and directional relationships. Second, the use of self-report measures may introduce several limitations. Social desirability bias may have led participants to overreport physical exercise and self-control while underreporting problematic smartphone use and academic burnout. Recall bias may have affected the accuracy of reported exercise behaviors. Additionally, common method variance may have inflated the observed correlations, as all variables were measured via self-report from the same source. Future studies could incorporate objective behavioral data (e.g., accelerometers for physical exercise, screen time logs for smartphone use) or multi-informant assessments to enhance data validity and reduce method bias. Finally, academic burnout is only one indicator of academic outcomes. Future research should include other psychological variables to further examine the protective associations of physical exercise with college students’ academic performance.

## Conclusion

This study identified distinct subtypes of physical exercise among college students, which differ in self-control, problematic smartphone use, and academic burnout. Moreover, the indirect associations of these subtypes with academic burnout via self-control and problematic smartphone use vary accordingly. Given the cross-sectional design of this study, all findings should be interpreted as associations rather than causal relationships. The observed patterns indicate that educators and policymakers may consider avoiding one-size-fits-all interventions and instead adopt tailored support strategies based on students’ specific exercise profiles. For occasional exercisers, interventions that enhance the enjoyment and accessibility of physical exercise may be associated with greater motivation to participate and the gradual establishment of regular exercise habits, which correlate with higher self-control and lower problematic smartphone use. For developing and regular exercisers, efforts that reinforce exercise habits may show associations with better self-control, more mindful smartphone use, and, through the serial pathway of “self-control → problematic smartphone use,” lower levels of academic burnout. Differentiated strategies may be more closely associated with higher self-control, lower problematic smartphone use, and lower academic burnout. Future longitudinal or experimental research is needed to determine whether the observed associations reflect causal pathways.

## Data Availability

The raw data supporting the conclusions of this article will be made available by the authors, without undue reservation.
